# Clinical Impact of Blood Pressure Variability in Kidney Transplant Patients: A Systematic Review and Meta-Analysis

**DOI:** 10.3390/life15081271

**Published:** 2025-08-11

**Authors:** Mehmet Kanbay, Alexandru Dan Costache, Crischentian Brinza, Ozgur Aktas, Busra Z. Bayici, Sevde Odemis, Candan Genc, Alexandru Burlacu, Irina Iuliana Costache Enache, Andreea Simona Covic, Pantelis Sarafidis, Masanari Kuwabara, Adrian Covic

**Affiliations:** 1 School of Medicine, Koç University, Istanbul 34450, Turkey; mkanbay@ku.edu.tr (M.K.); oaktas20@ku.edu.tr (O.A.); bbayici19@ku.edu.tr (B.Z.B.); sodemis19@ku.edu.tr (S.O.); cgenc19@ku.edu.tr (C.G.); 2 Faculty of Medicine, “Grigore T. Popa” University of Medicine and Pharmacy, 700115 Iasi, Romania; crischentian.brinza@d.umfiasi.ro (C.B.); alexandru.burlacu@umfiasi.ro (A.B.); ii.costache@yahoo.com (I.I.C.E.); andreea.covic@gmail.com (A.S.C.); accovic@gmail.com (A.C.); 3 Institute of Cardiovascular Diseases “Prof. Dr. George I.M. Georgescu”, 700503 Iasi, Romania; 4 ”St. Spiridon” Emergency County Hospital, 700111 Iasi, Romania; 5 ”Dr. C.I. Parhon” Clinical Hospital, 700503 Iasi, Romania; 6 Department of Nephrology, Hippokration Hospital, Aristotle University of Thessaloniki, 52124 Thessaloniki, Greece; psarafidis11@yahoo.gr; 7 Division of Cardiovascular Medicine, Department of Medicine, Jichi Medical University, Shimotsuke 329-0498, Japan; kuwamasa728@gmail.com

**Keywords:** kidney transplant, chronic kidney disease, blood pressure profile, dipping profile, non-dipper, reverse dipper, nocturnal hypertension

## Abstract

*Background:* The association between blood pressure (BP) dipping profiles and kidney function among chronic kidney disease (CKD) patients has been well established within the literature, but studies conducted on kidney transplant (KT) patients remain limited. Individual KT studies have small sample sizes and conflicting results. Meta-analysis overcomes these limitations by pooling data to increase statistical power and provide robust clinical guidance. This meta-analysis systematically assesses the impact of BP patterns on KT and CKD populations, aiming to highlight improved BP management strategies in these populations. *Materials and methods:* A comprehensive search was conducted up to September 9th, 2024, using multiple electronic databases. *Results:* The current study included 7 studies with a total of 788 patients. KT recipients showed a higher prevalence of non-dipper blood pressure profile than CKD patients. Also, those with a dipper profile had a significantly higher estimated glomerular filtration rate (eGFR) compared to non-dippers and reverse dippers, implying better graft function. No significant differences were observed in acute rejection risk, proteinuria, renal resistive index, cholesterol, or triglycerides across blood pressure profiles. *Conclusions:* These findings reveal a high prevalence of non-dipping blood pressure profiles in KT and CKD patients, linked to worse renal and cardiovascular outcomes, while also highlighting the need for ambulatory blood pressure monitoring and tailored BP management strategies in these high-risk populations to potentially improve outcomes. However, the observational nature of available studies limits causal inference, and further prospective research is required to establish definitive therapeutic recommendations.

## 1. Introduction

Hypertension (HTN) is a major concern in both chronic kidney disease (CKD) and kidney transplant (KT) patients due to it being an independent risk factor for progression of CKD, the development of cardiovascular disease, all-cause mortality, and graft function loss in these populations [1,2,3].

Although the conventional management strategy of HTN in CKD and KT recipients revolved around the measurement of blood pressure (BP) in a clinical setting, office BP lacks the ability to give insights on circadian BP variation and short-term BP variability, which is associated with aggravated cardiovascular and renal outcomes [4].

Ambulatory blood pressure monitoring (ABPM) has thus paved its way as the gold standard clinical tool for the evaluation and management of hypertension, as it allows serial BP monitoring at specific time intervals throughout a 24 h period, capturing intra- and inter-individual variability [5,6]. In essence, ABPM provides accurate insights into the dipping phenomenon and nocturnal HTN while allowing the diagnosis of particular forms of HTN, such as white-coat and masked HTN [7].

Normally, a healthy individual exhibits a 10% nocturnal decrement in BP values and is classified as a dipper. The non-dipping BP pattern corresponds to the failure of BP to decline during nighttime sleep by 10%. The pathophysiology behind the non-dipping BP phenomenon is dependent on myriads of mechanisms:

a. Disruption of the autonomic nervous system;
b. Disruption of the circadian rhythm;
c. Water and sodium dysregulation;
d. Endocrine disorders (e.g., thyroid) [8,9].

Typically, the expected dipping pattern occurs due to decreased cardiac output and heart rate at night, while systematic vascular resistance may remain slightly elevated. The non-dipping pattern arises from a lesser decline in nocturnal cardiac output or an exaggerated rise in vascular resistance [8,9,10].

The circadian rhythm, regulated by the suprachiasmatic nucleus, influences BP values via hormonal controls like melatonin and the autonomic nervous system. Disruptions in the sleep–wake cycle, such as obstructive sleep apnea and autonomic dysfunction, contribute to a non-dipping BP pattern [11].

High salt intake, particularly in salt-sensitive individuals, affects sodium excretion and endothelial dysfunction, further aggravating the non-dipping variability [8,10].

Hypertension is an independent risk factor for allograft dysfunction and failure in KT patients [3]. Hence, the high cardiovascular risk inherent to the KT population makes identification and management of abnormal BP patterns critically important for long-term patient survival.

The aim of the present meta-analysis is to evaluate the impact of different blood pressure profiles on kidney graft and renal function in KT recipients compared to CKD patients by investigating the variations in estimated glomerular filtration rate (eGFR), acute rejection risk, renal resistive index (RRI), proteinuria, and lipid levels and potentially improve the management of BP control in these populations.

## 2. Materials and Methods

This systematic review and meta-analysis was conducted in alignment with the Reporting Items for Systematic Reviews and Meta-Analyses (PRISMA 2020 guidelines) [12]. The study is registered with PROSPERO.

### 2.1. Literature Search

An extensive literature search was performed to determine studies focusing on the variability of blood pressure measurements in patients with KT and CKD up to the 9 September 2024.

The literature search was conducted in electronic databases (PubMed, Cochrane Library, Scopus, Web of Science, and Ovid MEDLINE) through the following terms:

- ‘Nocturnal hypertension’;
- ‘Reverse dipping’;
- ‘Non-dipping’;
- ‘Kidney transplantation’;
- ‘Renal transplantation’;
- ‘Renal outcome’;
- ‘Decline in kidney function’;
- ‘Kidney allograft failure/loss’;
- ‘Cardiovascular event’;
- ‘All-cause mortality’.

In addition to the initial database search, a manual search was conducted to detect unidentified articles. All the studies gathered from both electronic and manual searches were transferred into Covidence for a more comprehensive assessment. All articles were evaluated by two authors and reviewed by a third reviewer in case of a conflict.

The search strategy and keyword combinations are shown in Table 1.

### 2.2. Study Selection Process

The primary database search included 965 studies from electronic databases and 40 studies that were identified through the manual search. After the removal of duplicates, 868 studies were screened for meta-analysis.

The inclusion criteria in this meta-analysis were as follows:

- Studies comprising adult patients age ≥18;
- Studies published in English;
- Studies reporting ABPM outcomes in CKD and KT patients;
- Randomized controlled trials and prospective and retrospective cohort studies.

The exclusion criteria were as follows:

- Studies involving pediatric populations or animals;
- Literature reviews;
- Case reports;
- Non-comparable observational studies.

### 2.3. Outcome Measures

The outcome measures were as follows:

- eGFR;
- BP profiles (dipping, non-dipping, and reverse dipping) recorded by ABPM in CKD and KT patients;
- Acute rejection episodes;
- RRI;
- 24 h proteinuria;
- Serum cholesterol and triglyceride levels.

### 2.4. Statistical Analysis

The random-effects meta-analysis model was utilized for data synthesis. For categorical outcomes, odds ratios (ORs) were calculated, and for continuous outcomes, standardized mean differences (SMDs) were utilized. Both outcome types were reported with 95% confidence intervals (CIs). Statistical significance was defined as a *p*-value < 0.05. For the heterogeneity assessment, I^2^ statistics were used, with values of 25%, 50%, and 75% indicating low, moderate, and high heterogeneity, respectively. All statistical analyses were conducted using STATA V16.0.

The quality of the relevant studies was assessed with the Newcastle–Ottawa Scale (NOS), which evaluates studies based on the parameters of selection, comparability, and outcome ascertainment on a scale of 0 to 8.

After a detailed evaluation, 7 studies were included in the meta-analysis. Table 2 demonstrates the details of these studies, including baseline characteristics of the participants, study design, outcome measures, and results (see Table 2).

## 3. Results

**Figure 1:**
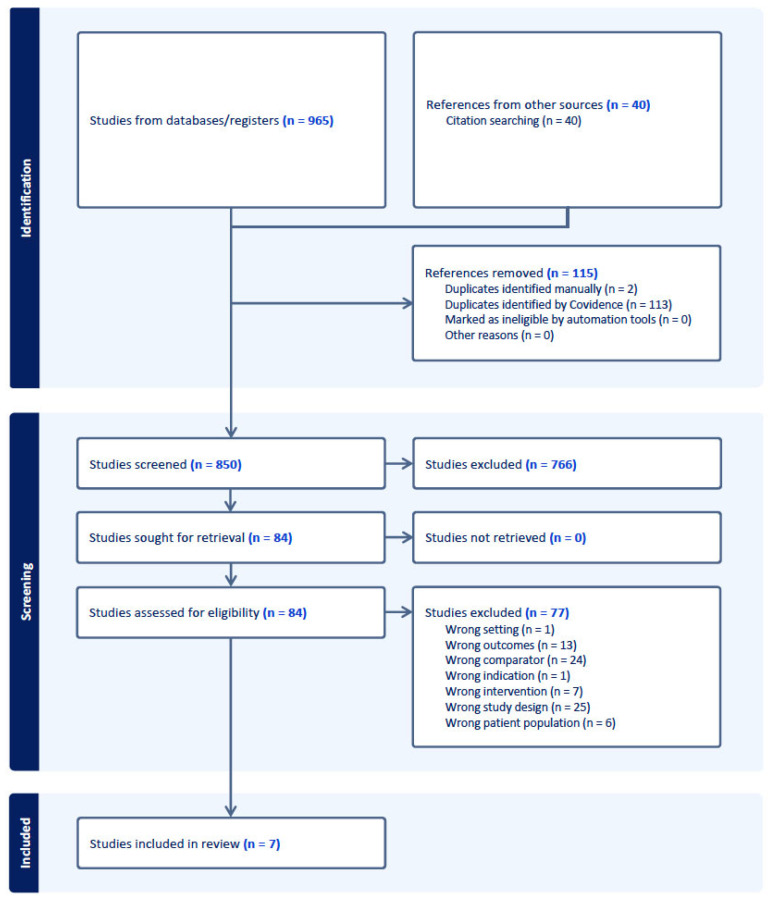
PRISMA flow diagram of study selection process.

The initial extensive search contained 965 studies; following the comprehensive search and post-screening procedure, 34 studies were included in the full-text review, of which 7 studies met all inclusion criteria and were included in the final meta-analysis (see Figure 1) [13,14,15,16,17,18,19].

Azancot et al. conducted the largest study, an observational cohort with 189 participants, consisting of 97 CKD and 92 KT patients [13]. In contrast, the study with the fewest participants was by Wajdlich et al., who included 55 participants, consisting of 41 CKD and 14 KT patients, in their prospective observational study [14]. Ibernon et al., in their prospective cohort study, had 126 patients, while Wadei et al. analyzed 119 participants in a retrospective cohort study design [15,16]. Similarly, Jaques et al. conducted a retrospective analysis with 123 patients, and Paoletti et al. included 95 KT recipients in their observational cohort [17,18]. Finally, Sezer et al. reported on 82 KT patients in their cross-sectional study [19].

### 3.1. Prevalence of Blood Pressure Patterns in CKD and KT Patients

**Figure 2:**
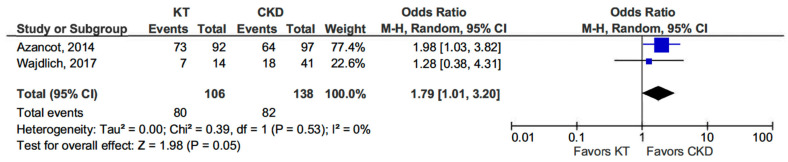
Forest plot of non-dipper profile in kidney transplant (KT) vs. chronic kidney disease (CKD) patients.

Two studies provided comparative data on blood pressure profiles in CKD patients and KT recipients. KT patients exhibited a higher prevalence of the non-dipper profile compared to those in the CKD group (OR 1.79, 95% CI, 1.01–3.20; *p* = 0.05) (see Figure 2) [13,14].

**Figure 3:**
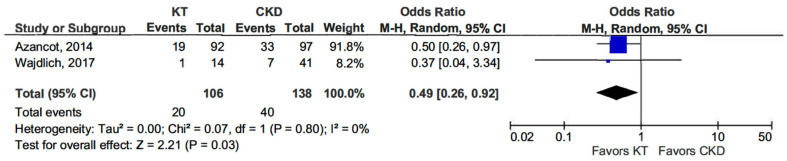
Forest plot of dipper profile in kidney transplant (KT) vs. chronic kidney disease (CKD) patients.

Additionally, the dipper profile was significantly less common in KT patients than in the CKD group (OR 0.49, 95% CI, 0.26–0.92; *p* = 0.03) (see Figure 3) [13,14].

### 3.2. Blood Pressure Profiles and Rejection Risk in KT Patients

**Figure 4:**
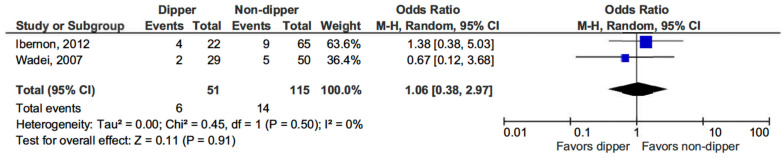
Forest plot of acute rejection episodes in dipper vs. non-dipper profile in kidney transplantation (KT) patients.

Regarding the risk of acute rejection in KT patients, no significant difference was observed between those with a dipping BP profile and non-dippers (OR 1.06, 95% CI, 0.38–2.97; *p* = 0.91) (see Figure 4) [15,16].

**Figure 5:**
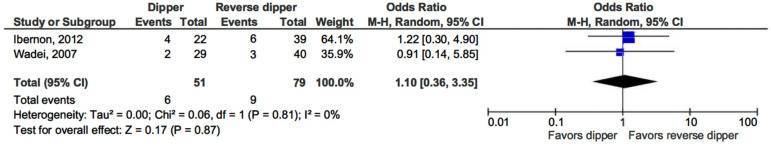
Forest plot of acute rejection episodes in dipper vs. reverse-dipper profile in kidney transplantation (KT) patients.

Similarly, the risk of acute rejection did not differ between patients with a dipping profile and reverse dippers (OR 1.10, 95% CI, 0.36–3.35; *p* = 0.87) (see Figure 5) [15,16].

### 3.3. Blood Pressure Profiles and Estimated Glomerular Filtration Rate in KT Patients

**Figure 6:**
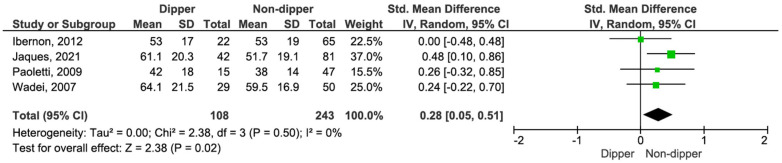
Forest plot of estimated glomerular filtration rate (eGFR) in dipper vs. non-dipper profile in kidney transplantation (KT) patients.

KT patients with a dipping profile exhibited a significantly higher eGFR compared to non-dippers (SMD 0.28, 95% CI, 0.05–0.51; *p* = 0.02) (see Figure 6) [15,16,17,18].

**Figure 7:**
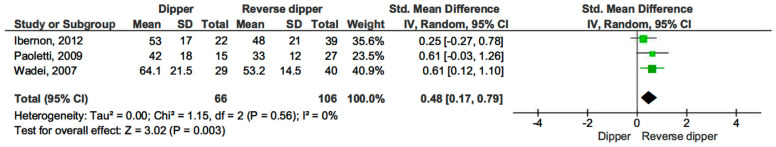
Forest plot of estimated glomerular filtration rate (eGFR) in dipper vs. reverse-dipper profile in kidney transplantation (KT) patients.

Additionally, dippers had improved eGFR values compared to those with a reverse-dipper profile (SMD 0.48, 95% CI, 0.17–0.79; *p* = 0.003) (see Figure 7) [15,16,17].

**Figure 8:**
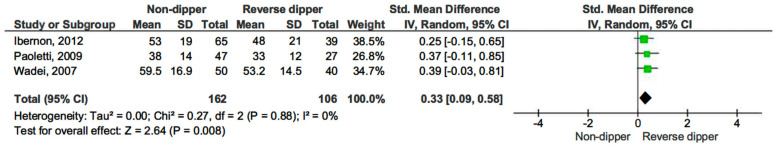
Forest plot of estimated glomerular filtration rate (eGFR) of non-dipper vs. reverse-dipper profile in kidney transplantation (KT) patients.

Non-dippers also displayed higher eGFR levels than reverse dippers (SMD 0.33, 95% CI, 0.09–0.58; *p* = 0.008) (see Figure 8) [15,16,17].

### 3.4. Blood Pressure Profiles and Proteinuria

**Figure 9:**
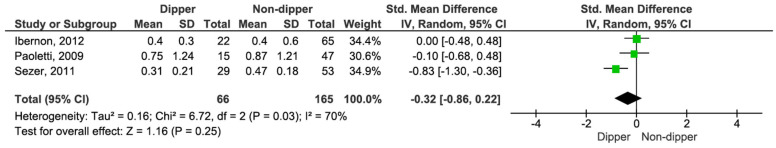
Forest plot of 24 h proteinuria in dipper vs. non-dipper in kidney transplantation (KT) patients.

**Figure 10:**
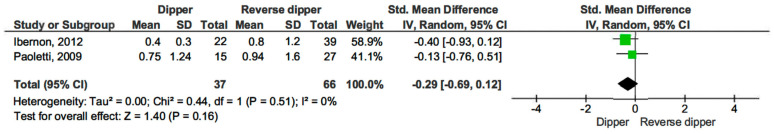
Forest plot of 24 h proteinuria in dipper vs. reverse dipper in kidney transplantation (KT) patients.

**Figure 11:**
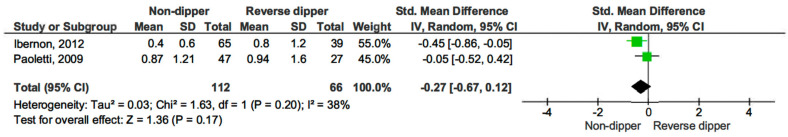
Forest plot of 24 h proteinuria in non-dipper vs. reverse dipper in kidney transplantation (KT) patients.

Additionally, we compared 24 h proteinuria across different blood pressure profiles. Proteinuria levels were similar across all comparisons: dippers versus non-dippers (SMD −0.32, 95% CI, −0.86 to 0.22; *p* = 0.25) (see Figure 9) [15,17,19], dippers versus reverse dippers (SMD −0.29, 95% CI, −0.69 to 0.12; *p* = 0.16) (see Figure 10) [15,17], and non-dippers versus reverse dippers (SMD −0.27, 95% CI, −0.67 to 0.12; *p* = 0.17) (see Figure 11) [15,17].

### 3.5. Blood Pressure Profiles and Renal Resistive Index

**Figure 12:**
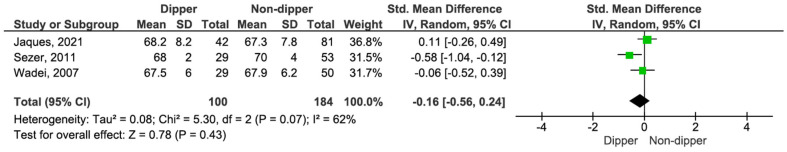
Forest plot of RRI in dipper vs. non-dipper in kidney transplantation (KT) patients.

Three studies provided comparative data on RRI between dipping and non-dipping profiles. RRI was similar across both blood pressure groups, with an SMD of −0.16 (95% CI, −0.56 to 0.24; *p* = 0.43) (see Figure 12) [16,18,19].

## 4. Discussion

This meta-analysis provides key insights into the prevalence and clinical impact of BP variability among KT recipients and CKD patients, reinforcing the critical role of ABPM in the identification of nocturnal hypertension patterns of dipping, non-dipping, and reverse-dipping BP variability in specifically KT and CKD patient populations. To reiterate our findings, KT patients had a higher prevalence of non-dipper BP profiles compared to CKD patients, highlighting the need to tailor BP management strategies in this category. The inverse association between kidney function and non-dipping status among CKD patients has been well established in multiple clinical trials, whereas studies conducted on kidney transplant recipients are scarce [20,21,22].

A meta-analysis evaluating a total of 4115 kidney transplant recipients from 42 clinical studies illustrated high rates of uncontrolled HTN (56%) according to ABPM and non-dipping status (54%, 95% CI: 45–63%) [2].

The study at hand also displayed that KT patients with a dipping profile had significantly higher eGFR values compared to their non-dipper and reverse-dipper counterparts, highlighting that maintenance of the circadian rhythm in BP may preserve graft function and viability. An analysis of 1.061 patients during 4.759 person-years of follow-up period illustrated that every 10% increase in nighttime systolic BP is linked to a 1.21-fold increase in CKD progression, defined as at least a 50% decline in eGFR or initiation of kidney replacement therapies [6].

This study has also provided the first strong evidence of a similar pattern of association between CKD progression and non-dipping blood pressure status. Another large-scale clinical trial conducted on over 906 patients with CKD stages 1 to 3 identified absence of nocturnal dipping as a risk factor for CKD progression, as patients without a nighttime dipping pattern are at higher risk for CKD progression even if they maintain 24 h ambulatory BP goals (HR 1.82, 95% CI: 1.17–2.82) [23].

Similarly, multiple other clinical trials have identified a non-dipping BP pattern as a risk factor for CKD progression among normotensive individuals [24,25,26]. It is also considered a cardiovascular mortality predictor and is associated with worse outcomes in hemodialysis patients [27].

In short, these findings suggest that non-dipping and reverse-dipper profiles are associated with poorer kidney graft function compared to dippers. This is, to the best of our knowledge, the first large-scale clinical data revealing higher rates of non-dipping status among KT recipients compared to the CKD population with low heterogeneity. Such an outcome indicates the presence of potentially irreversible pathophysiological events leading to non-dipping status and nocturnal HTN among CKD patients, which persist even in the post-transplant period despite improvements in kidney function.

Nevertheless, our meta-analysis lacks such analysis, highlighting a major limitation of our study. Our analysis revealed no significant difference in acute rejection rates between dipping and non-dipping profiles, implying that BP variability may impact graft function through chronic hemodynamic alterations rather than via immunologic rejection events. In addition, no notable differences were observed in proteinuria, RRI, or lipid levels across BP profiles, suggesting that the primary impact of a non-dipping profile is likely related to graft hemodynamics rather than acute renal damage or metabolic disturbances.

The exact underlying mechanism of non-dipping status among either CKD patients or KT recipients is unclear, though multiple hypotheses have been postulated. The following factors appear to be involved in the pathogenesis of a non-dipping BP pattern among CKD patients: disruption of circadian rhythm regulated by the suprachiasmatic nucleus of the hypothalamus mediated via upregulation of angiotensin-1 or uremic state; impaired sleeping pattern, including later onset, shorter duration, and increased fragmentation of sleep cycle; over-activation of the sympathetic nervous system and the renin–angiotensin–aldosterone system, leading to salt retention and vasoconstriction; and multiple comorbidities, including obstructive sleep apnea, diabetes mellitus, and obesity [28,29,30,31,32].

Post-transplant graft function and immunosuppressive regimens may be potential additional contributors to 24 h ABPM among KT recipients, though further pre-clinical and clinical studies are required for a better understanding of underlying molecular mechanisms [29].

This hypothesis was also brought into question by Mendoza-Romo-Ramírez et al. Their study found a higher frequency of non-dipping BP profiles in KT patients compared to the general population and proposed it to be a consequence of immunosuppressive therapy (cyclosporine-induced arterial hypertension), along with changes in homocysteine and alterations in the autonomic nervous system encountered in chronic renal patients [33]. Similar findings were previously presented by Lipkin et al. when comparing cyclosporin- and non-cyclosporin-treated renal transplant recipients [34].

Along with the recipients, living kidney donors should also be evaluated with ABPM, as shown by Yazawa et al. Although they are expected to have an increase in BP of around 5–10 mmHg, in their study, they did not show any significant increase in values. However, around 40% displayed non-dipping BP profiles following nephrectomy [35].

In CKD patients, the prevalence of resistant hypertension and abnormal BP profiles is also higher and requires investigation through 24 h ABPM and targeted pathophysiological treatment, i.e., optimizing diuretic regimens in volume excess assessment, renin–angiotensin–aldosterone system blockade in hyperactive states, or beta-/alpha-blockade in the presence of clues of sympathetic nervous system mediation [36].

Apart from the BP profile, ABPM can aid in identifying several specific forms of HTN, such as white-coat or sustained HTN. These are significant in renal patients, since they are also associated with histopathological alterations in kidney biopsies [37].

On the other hand, BP values vary significantly in CKD patients depending on the stages, and since consistency is poor, several publications are against using single 24 h ABPM measurements for properly assessing BP profile [38].

Our meta-analysis has provided insights into the association between 24 h ABPM and multiple clinical outcomes among kidney transplant recipients, though our study is not without major limitations. First, our meta-analysis includes data from only a few observational studies, potentially limiting the generalizability of our outcomes and potential alterations in outcomes with the publication of future studies. The observational design carries a risk of residual confounding despite statistical adjustments. Second, while statistical heterogeneity across the included studies appears low, the small number of studies (n = 7) and limited sample sizes for several comparisons restrict the statistical power and limit the robustness of pooled estimates. Third, we were unable to perform separate analysis on normotensive individuals with non-dipping status, which may provide valuable information regarding whether our clinical outcomes may solely be attributable to dipping pattern. Lastly, our meta-analysis lacks data on potential confounding factors on post-transplant blood pressure readings, including maintenance immunosuppression regimens, anti-hypertensive therapies, and comorbidities such as diabetes mellitus and metabolic syndrome. Due to the limited number of studies available, meaningful subgroup analyses to address these potential confounders were not feasible. Despite these limitations, the consistency of findings across diverse populations and study designs strengthens confidence in the observed association.

Although several studies have addressed the association between blood pressure variability and outcomes in either CKD or KT patients, the study at hand is the first comprehensive analysis to include both populations.

This meta-analysis at hand not only captures the high prevalence of non-dipping profiles among KT and CKD patients but also provides evidence on its significant implications for graft function and renal outcomes. Non-dipping and reverse-dipping profiles are linked to lower estimated glomerular filtration rate (eGFR) and potentially poorer graft function, while dipping profiles are correlated with better renal outcomes (see Table 3).

## 5. Conclusions

These findings emphasize the need for ABPM use in CKD patients and KT recipients to monitor nocturnal BP patterns. For reverse and non-dipper populations, personalized BP management strategies, such as sodium restriction, timely antihypertensive therapy, and lifestyle modifications, should be considered. The observational nature of the included studies limits causal inference, but the strength and consistency of associations across diverse populations support clinical relevance. These findings provide the best currently available evidence to inform clinical practice while recognizing the need for additional high-quality research, including prospective studies with standardized blood pressure monitoring protocols. Hence, more effective management of BP variability could enhance the quality of life and survival of these vulnerable groups.

## Figures and Tables

**Table 1:** Quality assessment of included studies using the Newcastle–Ottawa Scale for cohort studies.

Study	Study Design	Selection				Comparability		Outcome			Total Score
		*Representative of Exposed Cohort*	*Selection of Non-Exposed Cohort*	*Ascertainment of Exposure*	*Outcome Not Present at Start*	*Main Factor*	*Additional Factor*	*Assessment of Outcome*	*Follow-Up > 1 Year*	*Follow-Up Adequate*	
Azancot, 2014 [13]	Observational Cohort	*	*	*	*	*	*	*			**7**
Wajdlich, 2017 [14]	Prospective Observational	*	*	*	*	*		*			**6**
Ibernon, 2012 [15]	Prospective Cohort	*		*	*	*		*	*	*	**7**
Wadei, 2007 [16]	Retrospective Cohort	*		*	*	*	*	*	*	*	**8**
Paoletti, 2009 [17]	Observational cohort	*		*	*	*	*	*	*	*	**8**
Jaques, 2021 [18]	Retrospective cohort	*		*	*	*	*	*	*	*	**8**
Sezer, 2011 [19]	Cross-sectional	*		*	*	*	*	*			**6**

* it matches the criteria

**Table 2:** Characteristics of the included studies.

Study	Study Design	Follow-Up Duration	Number of Participants/Population Characteristics	Outcome Measures	Key Findings
Azancot et al. (2014) [13]	Observational cohort study	Cross-sectional data collection	- 189 participants CKD (n = 97) KTR (n = 92) - Mean age: 52.5 years - Gender: 71.1% male in CKD, 73.9% male in KTR - Mean BMI: 27.5 kg/m^2^ for CKD, 26.8 kg/m^2^ for KTR - Time after transplantation: mean 73.7 months	Office and 24 h ABPM: - Transplantation was identified as an independent predictor of higher nighttime BP, contributing to non-dipping and reverse-dipping patterns	- Office BP was similar between kidney transplants and CKD patients. - ABPM showed that abnormal dipping patterns in KTR, particularly reverse dipping, were associated with higher nighttime systolic BP. - Transplants had a higher prevalence of nocturnal hypertension and non-dipper BP patterns, linked to increased cardiovascular risk. - Transplantation was an independent predictor of higher nighttime BP, contributing to non-dipping and reverse-dipping patterns. - ABPM is recommended over office BP for accurate hypertension assessment in transplant patients.
Wajdlich et al. (2017) [14]	Prospective observational study	Cross-sectional data collection	- 55 participants: CKD (n = 41) KTR (n = 14) - Mean age: CKD patients, 67 years; KTR, 53 years - Gender: CKD, 46% male; KTR, 57% male - BMI: CKD, 24 ± 5 kg/m^2^; KTR, 25 ± 3 kg/m^2^ - eGFR: mean 40.1 mL/min in CKD; 46.4 mL/min in KTR	- 24 h ABPM - Urinary sodium excretion measured during daytime and nighttime activities	- Non-dipping and reverse dipping were prevalent among KTR and CKD patients; normal dipping was rare. - In both groups, non-dipping and reverse-dipping patterns were associated with impaired kidney function, indicated by lower eGFR levels. - There was a strong inverse correlation between nighttime BP fall and the night-to-day natriuresis ratio, indicating impaired natriuresis may contribute to abnormal dipping. - ABPM is clinically relevant for identifying abnormal dipping profiles linked to higher cardiovascular and renal risk.
Ibernon et al. (2012) [15]	Prospective cohort study	45 ± 11 months	- 126 patients (87 male, 39 female) - Patient age: 53 ± 13 years (dippers) - 52 ± 13 years (non-dippers) and 54 ± 13 years (reverse dippers) - BMI: higher in reverse dippers (26.9 ± 5.0 kg/m^2^) compared to others - Post-transplant clinical outcomes at 3 months Acute rejection episodes: dippers, 4 patients (18%); non-dippers, 9 patients (14%); reverse dippers, 6 patients (15%)	- ABPM - CrCl - Proteinuria - Graft failure	In RTRs, the reverse-dipping pattern was associated with renal target organ damage and an increased left ventricular mass, which are important and common risk factors for graft loss and CV events.
Wadei et al. (2007) [16]	Retrospective cohort study	1-year post-transplantation	- 119 pt. - 62% male - Age: 51.4 - Age (year and median range): 51.4 (17 to 79) - Male gender (n%): 74 (62) - White race (n%): 117 (98) - Pre-transplantation BMI (kg/m^2^ mean SD): 27.4 5.0	- ABPM - GFR - RRI - Biopsy	* Non-dippers and reverse dippers had significantly lower GFR compared to dippers (*p* = 0.04). * There was a correlation between greater nocturnal drop in systolic BP and better allograft function, with GFR increasing by 4.6 mL/min for every 10% nocturnal drop in systolic BP. * Elevated RI and loss of nocturnal BP fall were both independent predictors of lower GFR, suggesting that abnormal diurnal BP patterns relate to poor kidney transplant outcomes.
Paoletti et al. (2009) [17]	Observational cohort study	1-year post-transplantation	- Participants: 94 renal transplant recipients (RTRs) - Mean age: 55 - Gender: 69% male - BMI: mean 24.8 ± 4.4 kg/m^2^ - eGFR: mean 38 ± 14 mL/min	- 24 h ambulatory blood pressure monitoring (ABPM) - Serum creatinine and daily urinary protein excretion	- Only 5% of KTR met the target blood pressure of 130/80 mm Hg, with a high occurrence of abnormal dipping patterns, particularly non-dipping and reverse dipping. - Non-dipping and reverse dipping were associated with higher serum creatinine and proteinuria levels, suggesting a connection between abnormal dipping status and kidney function. - Awake systolic BP predicted daily proteinuria, while asleep diastolic BP was linked to serum creatinine, highlighting specific blood pressure parameters related to renal outcomes and emphasizing the need for ABPM to identify high-risk patients.
Jaques et al. (2021) [18]	Retrospective cohort study	2 years after the initial ABPM measurement, with visits at 1 year and 2 years post- ABPM	- 123 pt. - Age: 56.0 ± 15.1 years - Baseline eGFR: 54.9 ± 20.0 mL/min - Non-dipper: 65.8%	- eGFR - ABPM	* Non-dippers had significantly lower eGFR compared to dippers at baseline (mean eGFR of 51.7 mL/min/1.73 m^2^ vs. 61.1 mL/min/1.73 m^2^ in dippers). * Non-dippers showed a faster decline in eGFR compared to dippers * Dippers had a 10.4 mL/min/1.73 m^2^ higher eGFR compared to non-dippers, independent of confounders such as hypertension and proteinuria. * Hypertension negatively affected renal function (*p* = 0.003).
Sezer et al. (2011) [19]	Cross-sectional study	Cross-sectional data collection	- Participants: 82 KTR - Mean age: 37.3 ± 10.8 years - Gender: 67% male - BMI: mean 27.6 kg/m^2^ - Time after transplantation: mean 50–52 months	- 24 h ABPM - RRI assessed via Doppler ultrasonography	- 65% of patients exhibited a non-dipping blood pressure pattern, which was strongly associated with increased RRI and higher levels of CRP, uric acid, and proteinuria. - Increased RRI (≥0.7) was identified as an independent predictor of non-dipping status, indicating a link to impaired renal function. - Non-dipping hypertension was associated with worse renal outcomes, underscoring the role of ABPM in identifying high-risk patients who may benefit from intensified blood pressure management strategies aimed at improving graft survival and reducing cardiovascular events.

KTR: kidney transplant recipient, BP: blood pressure, ABPM: ambulatory blood pressure monitoring, BMI: body mass index, RRI: renal resistive index, CRP: C-reactive protein, eGFR: estimated glomerular filtration rate, CrCL: creatinine clearance, CKD: chronic kidney disease.

**Table 3:** What is known and unknown regarding the dipping status of blood pressure among kidney transplant recipients.

**What Is Known?**
✓ Hypertension is a major comorbidity among kidney transplant recipients and is associated with poor clinical outcomes, including cardiovascular diseases, graft functional decline or loss, and all-cause mortality. ✓ Non-dipping or reverse-dipping pattern is associated with worse clinical outcomes, even among normotensive patients. ✓ Non-dipping or reverse-dipping pattern is mediated via disruption of the autonomic nervous system and circadian rhythm, water and salt homeostasis, and endothelial dysfunction.
**What Is Unknown?**
✓ KT recipients have a higher prevalence of non-dipping or reverse-dipping patterns compared to chronic kidney disease patients. ✓ KT recipients with a non-dipping or reverse-dipping pattern for BP have worse graft function, illustrated via lower estimated glomerular filtration rates compared to patients with a dipping pattern. ✓ Dipping status of blood pressure among kidney transplant recipients is not associated with rejection episodes, 24 h proteinuria, renal resistive index, or lipid profile.

## Abbreviations

The following abbreviations are used in this manuscript:

ABPM	Ambulatory blood pressure monitoring
BMI	Body mass index
BP	Blood pressure
CI	Confidence intervals
CKD	Chronic kidney disease
CrCl	Creatinine clearance
CRP	C-reactive protein
eGFR	Estimated glomerular filtration rate
HTN	Hypertension
KT	Kidney transplant
OR	Odds ratio
RRI	Renal resistive index
SMD	Standardized mean differences
